# Cardiomyocyte regeneration therapy and its effect on LVEF and scar size- a systematic review and meta-analysis

**DOI:** 10.1186/s13287-025-04357-8

**Published:** 2025-09-02

**Authors:** Mahnoor Mahnoor, Syed Irtaza Hassan, Muhammad Bakhtiar, Salal Sharjeel, Roha Daneyal, Muhammad Ibrahim, Raheel Iftikhar

**Affiliations:** 1 Armed Forces Bone Marrow Transplant Center, Rawalpindi, Pakistan; 2 Mohtarma Benazir Bhutto Shaheed Medical College, Mirpur, Pakistan; 3 Thomas Jefferson University- Sydney Kimmel Comprehensive Cancer Research Center, Philadelphia, USA; 4 Dow Medical College, Karachi, Pakistan; 5 King Edward Medical University, Lahore, Pakistan

**Keywords:** Cardiomyocyte regeneration, Stem cell therapy, Myocardial infarction, Left ventricular ejection fraction, Ischemic heart failure, Scar size

## Abstract

**Background:**

Myocardial infarction (MI) results in loss of cardiomyocytes leading to heart failure. Despite advancements in pharmacotherapy and interventions such as revascularization, ischemic heart failure remains a challenge. Recent advancements in stem cell therapies, genetic engineering and bioengineering have shown to improve cardiac function and quality of life.

**Methodology:**

Following PRISMA guidelines, randomized controlled trials clinical trials from last 12 years were systematically reviewed. All the patients included in these studies had ischemic heart failure and were subjected to different types of stem cell therapies. Protocol for this meta-analysis is registered on PROSPERO (Registration no: CRD42023399263). Data extraction and Quality assessment was done according to Cochrane handbook of systematic reviews and meta-analysis. Meta-analysis was conducted using Revman, and a random-effect model was used to calculate weighted mean differences (WMD) in left ventricular ejection fraction (LVEF), scar size and Minnesota Living with Heart Failure score (MLHFQ) pre- and post-intervention.

**Results:**

The pooled mean difference (MD) for scar size reduction at 6 months follow-up was − 0.36; (95%CI [-0.63, -0.10]), I^2^ = 71% (*p* < 0.0001) and at 12 months follow-up was − 0.62; (95%CI [-1.03, -0.21]), I^2^ = 78% (*p* < 0.0001) with a positive effect direction. Weight of the studies ranged from 5.4 to 10.8% and 9.6–14.1% at 6 months and 12 months follow-up respectively. The pooled data analysis at 6 months and 12 months follow-up revealed weighted mean difference 0.44; (95% CI [0.13–0.75]), I^2^ = 85% (*p* < 0.00001) and 0.64; 95% CI [0.14–1.14], I^2^ = 85% (*p* < 0.00001) respectively. For MHLFQ score pooled weighted mean difference was calculated for 286 patients which revealed mean difference − 0.38, (95% CI [-0.71-0.05]) (*p* = 0.02), I^2^ = 69% (*p* < 0.002). Sensitivity analysis by excluding ‘Gujjaro et al. 2016’ revealed weighted mean difference − 0.49; (95% CI [-0.74-0.25]) (*p* < 0.0001), I^2^ = 72% (*p* = 0.09).

**Conclusions:**

Our meta-analysis not only demonstrated consistent improvements in LVEF and reductions in scar size but also improvement in quality of life with stem cell therapies, however, the heterogeneity among studies calls for a need of standardized protocols and further research in optimizing these therapies to improve cardiomyocyte regeneration and overall cardiac repair.

## Introduction

Cardiovascular disease is one of the commonest causes of morbidity and mortality with acute myocardial infarction (MI) being a major contributor to heart failure [1]. An MI induces substantial loss of cardiomyocytes, resulting in the creation of non-contractile scar tissue. This remodeling leads to decreased left ventricular ejection fraction (LVEF) that negatively impacts cardiac function and longer-term patient outcome [2]. Conventional treatments like pharmacotherapy and device-based interventions are limited to treating symptoms or secondary prevention. However, they do not restore the loss of cardiomyocytes at the root; hence, there is a demand for novel therapeutic approach [3].

Studies have shown improvement in Cardiac structure and function through cardiomyocyte regeneration therapies. These therapies seek to either stimulate the endogenous regenerative capacity of the myocardium or bring in exogenous cells or molecules to replace the missing cardiomyocytes. Strategies such as mesenchymal stem cell therapy, cardiac tissue engineering, and gene therapy have been widely studied since the start of this century [4]. In the preclinical models, it has been reported that regenerative interventions are associated with enhanced LVEF, reduced infarct size, and the reduced amount of scar formation [5]. However, clinical trials have yielded inconsistent results, with few of the clinical trials reporting that the improvement in cardiac function was minimal or negligible [6]. These mixed findings raise critical questions regarding the efficacy and safety of cardiomyocyte regeneration therapies.

This review aims at meta-analyzing the effects of different types of stem cell therapies on cardiac function and quality of life in post MI patients with reduced left ventricular ejection failure (LVEF). All the preclinical and clinical evidence to further clarify the potential therapeutic efficacy and limitations of these interventions is included in this analysis.

## Methods

This systematic review and meta-analysis were conducted according to PRISMA guidelines [7].

### Eligibility criteria

We only included randomized control trials and clinical trials from the last 12 years in this systematic review and meta-analysis. All the selected articles had a defined outcome measure related to the effect of cardiomyocyte regeneration therapies in patients who have had at least one episode of myocardial infarction in the past. All the patients were > 18 years old whereas there was no restriction for gender, ethnicity or country. Only articles in English language were included. All the observational studies and duplicates were excluded along with the studies that had unreliable data.

### Information sources

A thorough search through PubMed, Embase, Cochrane library and clinical trials.gov was done.

### Search strategies

Different combinations of words for the terms like “Cardiomyocytes”, “Stem Cells”, “mesenchymal stem cells”, “cardiac derived stem cells”, “bone marrow stem cells”, “induced pluripotent stem cells” “Left Ventricular Ejection Fraction”, “Scar Size” and “Myocardial Infarction” were used. An expert was consulted to aid in searching process. The relevance of the search term was determined by its relevance with the research question and the number of articles yielded by the term. Search terms that gave irrelevant results were changed to improve search outcomes. Boolean operators like “AND” and “OR” were used to improve the search results. Wildcard operators “*/#” were used to bring up different word spellings of a single word. Search filters for RCT, CT, and English language were chosen to keep the search relevant.

### Selection process

The selected studies were first uploaded to Rayyan.ai. The built-in duplicate detection software in Rayyan was used to exclude duplicates. After deduplication, the titles and abstracts of the studies were screened against the inclusion and exclusion criteria. After initial screening, the remaining studies were subjected to a full text review to look for any excluding factors. Only studies meeting the inclusion criteria were included in this systematic review and meta-analysis. To minimize bias, 2 authors independently reviewed the studies during the selection process. Any disagreements between them were resolved by a 3rd author.

### Data items

The primary data outcomes our study targeted include LVEF and myocardial scar size before and after stem cell therapy in post MI patients at 6 month and 12-month follow-up within the same group. Other data that was extracted included sample size, median age, type of stem cells infused, mode of infusion and Minnesota Living with Heart Failure Questionnaire (MLHFQ) score before and after stem cell therapy [8].

### Risk of bias assessment

Risk of Bias (RoB 2.0) tool by RevMan 5.4 was used for quality assessment of randomized controlled trials with a control group [9]. Interventional studies without any control group were assessed for quality using NIH-NHLBI quality assessment questionnaire [10]. This questionnaire provides a checklist of 10 questions to help assess the quality of each individual study. Quality assessment was carried out by 2 authors for each individual study.

### Data synthesis

The eligibility of the studies for meta-analysis was determined by the presence of similar outcomes and data metric used in each study. Studies with sample size, mean, and SD values at either 6 months, 12 months or both were all selected for the meta-analyses.

The meta-analysis was conducted in Revman 5.4. A random-effect-model was used to calculate pooled effect size for all the outcomes having changes from the baseline. For all the studies, individual and pooled effects were presented in the form of forest plots. Relevant statistics for each study such as 95% confidence interval {CI} and weight of each study were also included in the forest plot. *I*^*2*^ was used to assess heterogeneity. The sensitivity of the analysis to individual studies was assessed by excluding each study and re-evaluating the results.

## Results

### Study selection

**Fig. 1:**
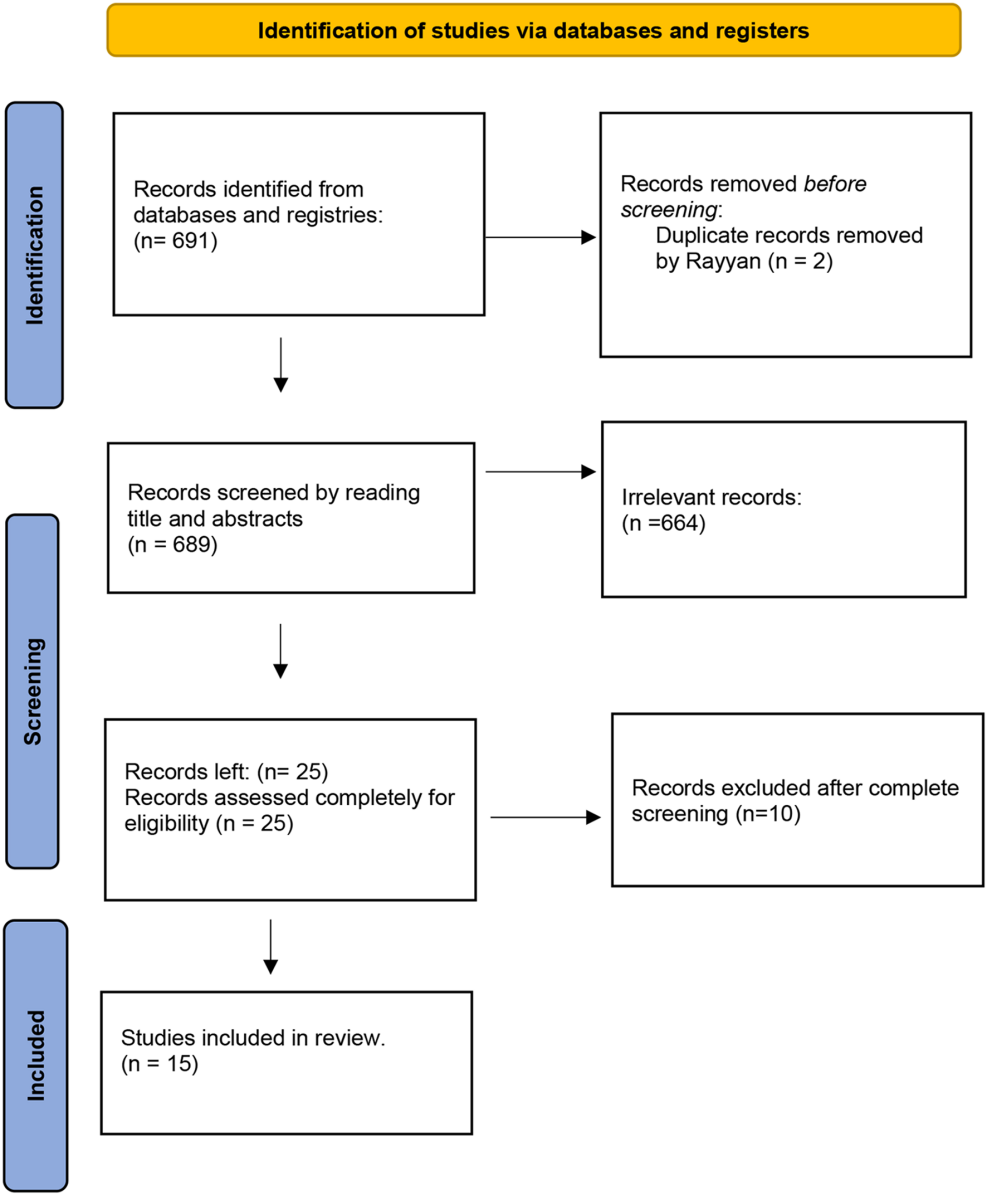
Flowchart indicating the selection of articles through the PRISMA method

A total of 691 studies were imported to our rayyan library. After the initial screening that involved deduplication and removal of irrelevant articles by reading titles and abstracts, 25 articles were left. A secondary screening which involved a full-length review of these articles was done and based on the eligibility criteria and 15 studies were included for quantitative analysis (see Fig. 1).

### Study characteristics

The study characteristics of 15 studies included in this systematic review and meta-analysis are shown in Table 1.

**Table 1:** Study characteristics of the cohort

Study ID and Publication Year	Study Design	Sample size	Intervention group	Control group	Median age	Type of Stem Cells	Mode of infusion
Anastasiadis et al. 2016 [11]	Phase 2 interventional	11	11	-	58 +/- 6.7	Mesenchymal precursor cell	Intra myocardial injection
SCIPIO Trial 2012 [12]	Nonrandomized interventional	33	20	13	57 +/- 3.1 for cases 55.8 for controls	Autologous cardiac sphere derived cells	Intra coronary injection
CADUCEUS Trial 2012 [13]	RCT	31	17	8	54 +/- 2.5 for cases 50.9 +/- 5.5 for controls	Cardiosphere derived cells	Intra coronary injection
MESAMI 1 Trial 2017 [14]	Single arm interventional	13	13	-	61.8 +/- 4	Skeletal muscle derived stem cells	Endomyocardial injection
CADUCEUS Trial 2014 [15]	Interventional RCT	31	23	8	-	Cardiosphere derived cells	Intra coronary injection
ALLSTAR Trial 2020 [16]	RCT	142	99	44	55 +/- 11 for cases 54 +/- 10 for controls	Cardiosphere derived stem cells	Intra coronary injection
ALLSTAR Trial 2021 [16]	Randomized clinical trial	142	95	47	53.5 +/- 10.2 for cases 54.7 +/- 11.1 for controls	Cardiosphere derived stem cells	-
MESAMI 1 Trial 2016 [17]	Open label interventional	10	10	-	-	Bone marrow-derived mesenchymal stromal cells	Intramyocardial
Li et al. 2014 [18]	Randomize d open label	69	33	36	MSC group: 53.9+/-10.5 Control: 54.2+/-77.7	Bone marrow-derived mesenchymal stromal cells	Intracoronary
CONCERT-HF Trial 2021 [19]	Double blind placebo controlled, phase 2 trial	125	33/125	32	61.0+/-11.1	Mesenchymal stromal cells and c-kit positive cardiac cells	Transendocardial injection
IMPACT-CABG Trial 2016 [20]	Phase 2 randomized clinical trial	40	19	20	65.2+/-7.2	Bone Marrow derived mononuclear cells	Intramyocardial injection
SWISS-AMI Trial 2016 [21]	Randomized control trial	200	66	67	-	Bone Marrow derived mononuclear cells	Intracoronary
Danish Phase II 2023 [22]	Double-blind, placebo control, phase 2	81	54	Isotonic saline (*n* = 27)	67	Adipose tissue derived mesenchymal Stromal Cell	Intra-myocardial injection
SCIENCE Trial 2023 [23]	Double-blind, placebo control, phase 2	133	90	43	ASC: 66.4+/-8.1, Placebo 64.0+/-8.8)	Adipose tissue derived mesenchymal Stromal Cell	Intra-myocardial injection
PRECISE Trial 2014 [24]	Randomized, placebo controlled, double-blind trial	27	21	06	63.6+/-7.5	Adipose tissue derived regenerative cells	Transendocardial

### Quantitative analysis

#### Reduction in scar size

**Fig. 2:**
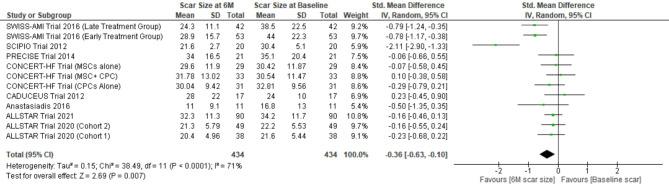
Forest plot representing changes in myocardial scar size at 6 months follow-up (6 M: 6 months, scar size at baseline: scar size before stem cell therapy)

**Fig. 3:**
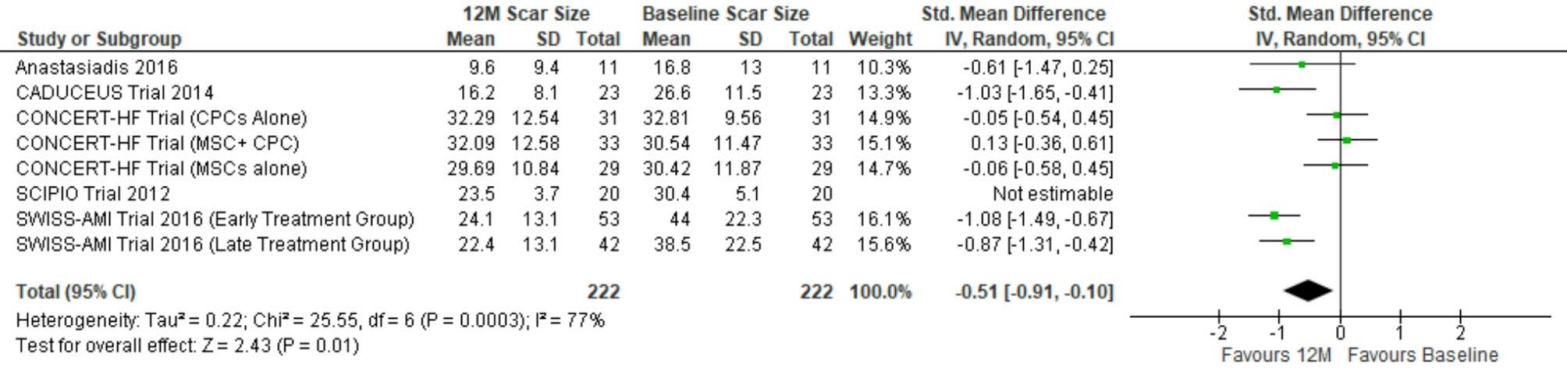
Forest plot representing changes in myocardial scar size at 12 months follow-up (12 M: 12 months, scar size at baseline: Scar size before stem cell therapy)

The pooled weighted mean difference for scar size reduction at 6 months follow-up calculated for 434 patients showed − 0.36; (95%CI [-0.63, -0.10]), I^2^ = 71% (*p* < 0.0001) and for 242 patients at 12 months follow-up was − 0.62; (95%CI [-1.03, -0.21]), I^2^ = 78% (*p* < 0.0001) with positive effect direction. Weight of the studies ranged from 5.4 to 10.8% and 9.6–14.1% at 6 months and 12 months follow-up respectively. Sensitivity analysis was done by excluding ‘SCIPIO trial’ and scar size further reduced to -0.26; (95%CI [-0.46, -0.07]) (*p* = 0.008), I^2^ = 45% (*p* = 0.05) and − 0.51 (95% CI [-0.91, -0.10]) (*p* = 0.01), I2 = 77% (*p* = 0.0003) at and 12 months follow-up respectively. (Figs. 2 & 3).

#### Improvement in LVEF

**Fig. 4:**
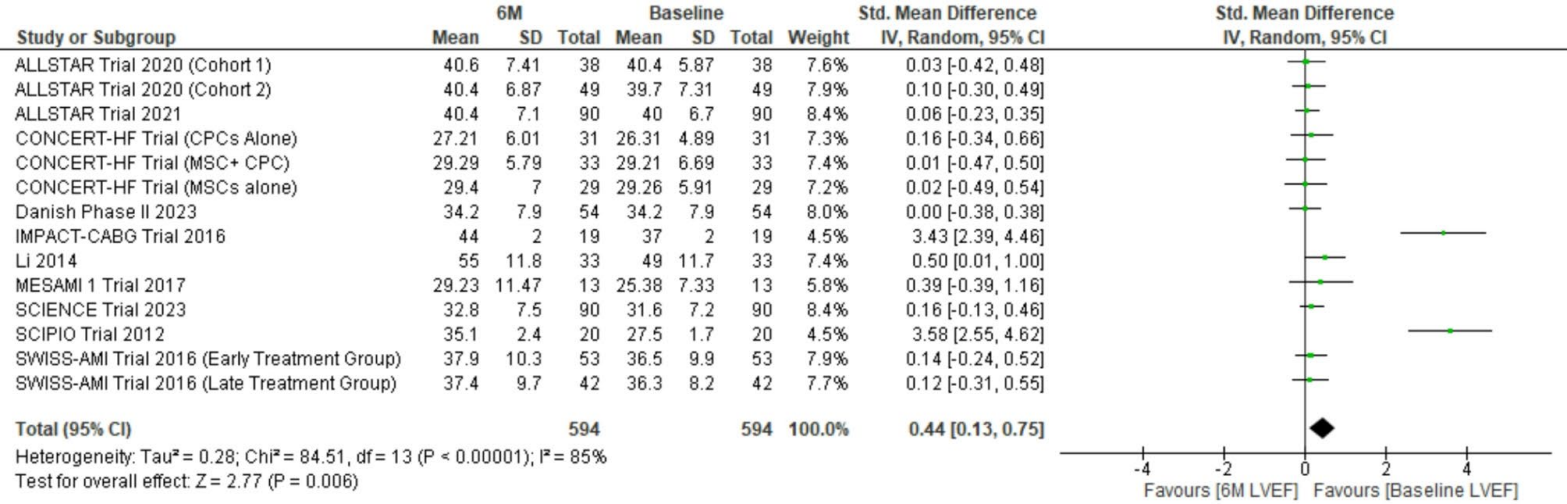
Forest plot representing changes in LVEF at 6 months follow-up (LVEF: Left ventricular ejection failure, 6 M LVEF: LVEF at 6 months after stem cell therapy)

**Fig. 5:**
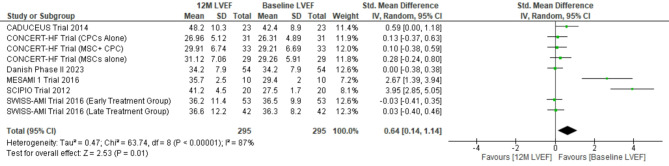
Forest plot representing changes in LVEF at 12 months follow-up (12 M LVEF: LVEF at 12 months follow-up)

The pooled weighted mean difference for 594 patients at 6 months and 295 patients at 12 months follow-up revealed weighted mean difference 0.44; (95% CI [0.13–0.75]) (*p* = 0.006), I^2^ = 85% (*p* < 0.00001) and 0.64; 95% CI [0.14–1.14] (*p* = 0.01), I^2^ = 87% (*p* < 0.00001) respectively. Sensitivity analysis by excluding SCIPIO trial revealed weighted mean difference 0.25; (95% CI [0.01–0.48]) (*p* = 0.04), I^2^ = 72% (*p* < 0.0001) and 0.25; (95% CI [0.05–0.54]) (*p* = 0.10), I^2^ = 64% (*p* = 0.008) at 6 and 12 months respectively (Figs. 4 and 5).

#### Minnesota living with heart failure questionnaire (MHLFQ) score

**Fig. 6:**
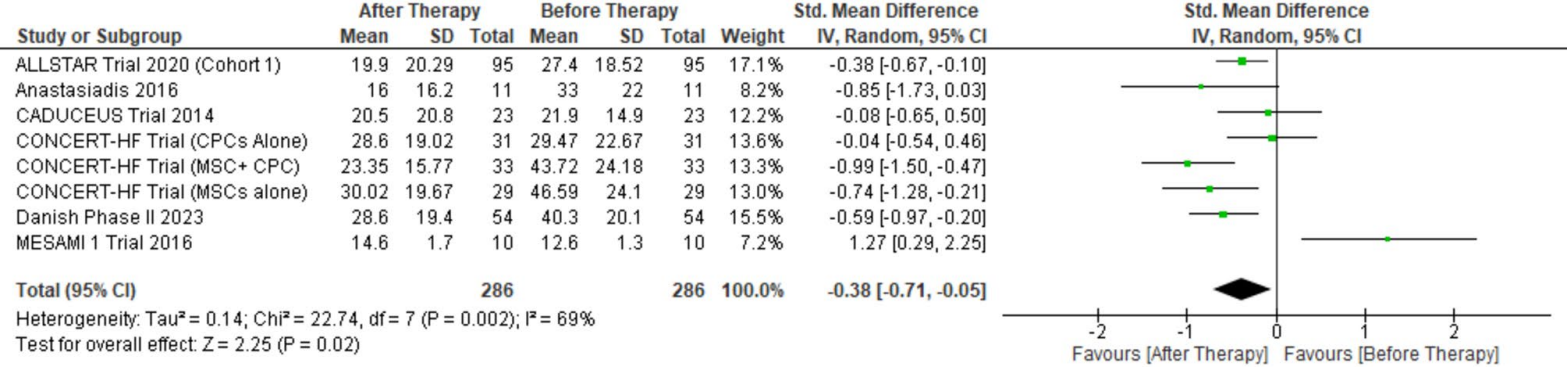
MHLFQ after stem cell therapy. (MHLFQ Score: Minnesota Living with heart failure questionnaire (MHLFQ) score)

For MHLFQ score pooled weighted mean difference was calculated for 286 patients which revealed mean difference − 0.38, (95% CI [-0.71-0.05]) (*p* = 0.02), I^2^ = 69% (*p* < 0.002). Sensitivity analysis by excluding ‘MESAMI 1 Trial 2016’ revealed weighted mean difference − 0.49; (95% CI [-0.74-0.25]) (*p* < 0.0001), I^2^ = 72% (*p* = 0.09) (Fig. 6).

#### Quality assessment of studies

**Fig. 7:**
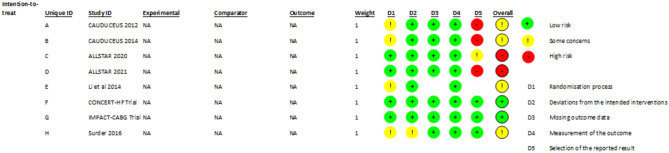
RoB quality assessment of studies

For the 11 randomized control trials the Rob tool was used to evaluate randomization, allocation concealment, selective reporting, blinding and incomplete outcome data. Of these, five studies had low risk of bias, five had some concerns whereas one study had high risk of bias (Fig. 7).

NHLBI quality assessment tool was used to determine the quality of the remaining studies. Based on this evaluation 3 studies were found to be of good quality and one study had moderate quality with some limitations (Table 2).

**Table 2:** NHLBI quality assessment of studies

NIH criteria	SCIPIO Trial 2012	Anastasiadis 2016	MESAMI 1 2017	MESAMI 1 2016
Was the study question or objective clearly stated?	Y	Y	Y	Y
Were eligibility/selection criteria for the study population prespecified and clearly described?	Y	Y	Y	Y
Were the participants in the study representative of those who would be eligible for the test/service/intervention in the general or clinical population of interest?	Y	Y	Y	CD
Were all eligible participants that met the prespecified entry criteria enrolled?	Y	Y	Y	Y
Was the sample size sufficiently large to provide confidence in the findings?	N	Y	CD	CD
Was the test/service/intervention clearly described and delivered consistently across the study population?	Y	Y	Y	Y
Were the outcome measures prespecified, clearly defined, valid, reliable, and assessed consistently across all study participants?	Y	Y	Y	Y
Were the people assessing the outcomes blinded to the participants’ exposures/interventions?	N	Y	CD	CD
Was the loss to follow-up after baseline 20% or less? Were those lost to follow-up accounted for in the analysis?	Y	Y	CD	CD
Did the statistical methods examine changes in outcome measures from before to after the intervention? Were statistical tests done that provided p values for the pre-to-post changes?	Y	Y	Y	Y
Were outcome measures of interest taken multiple times before the intervention and multiple times after the intervention (i.e., did they use an interrupted time-series design)?	Y	Y	Y	Y
If the intervention was conducted at a group level (e.g., a whole hospital, a community, etc.) did the statistical analysis consider the use of individual-level data to determine effects at the group level?	-	-	-	-
Quality	Moderate	High	Good	Good

## Discussion

To the best of our knowledge, this is the first meta-analysis that exclusively includes human population to assess the efficacy of cardiomyocyte regeneration therapies in patients with ischemic heart failure. Our meta-analysis, which includes 11 RCTs and 4 interventional studies demonstrates that stem cell-based therapies show slight improvement in LVEF and reduction in scar size but significant improvement in quality of life as indicated by improvement in MHLFQ scores after therapy. Most of the studies in our review were of high quality, which indicates the reliability of evidence explained in this review. However, there was one high risk study and a few studies with some concerns which were considered in the interpretation of results and further explored by sensitivity analysis where applicable. Sensitivity analysis was done by excluding ‘SCIPIO trial’ and ‘MESAMI 1 TRIAL” for scar size and LVEF respectively but it did not lead to any significant changes in outcome. Our findings are consistent with the meta-analysis conducted by Fischer et al. 2015 [25] which includes patients with heart failure, however, our study exclusively focuses on post-MI patients.

The first-generation stem cells including BMSCs and MSCs showed variable efficacy across clinical trials. While trials such as TOPCARE-AMI [26], REPAIR-AMI [25] and BOOST [27] demonstrated improvements in LVEF, others such as ASTAMI [28] and HEBE [29] did not show any significant changes. This heterogeneity in results can be due to differences in timing of administration, route of administration, patients’ comorbidities and variation in cell viability. Clinical trials demonstrate that MSCs derived from different sources such as bone marrow, umbilical cord or adipose tissue can improve cardiac function [30]. MSCs were initially considered immune-privileged, however, recent evidence challenges this notion which raises questions about their efficacy and engraftment [31, 32]. Pre-clinical trials demonstrated better outcomes with BM derived MSCs due to their trilineage differentiation [33]. POSEIDON trial (NCT01087996) investigated the efficacy of MSC’s delivered through trans-endocardial route and the findings suggested that low doses of MSC’s have better outcomes. It can be because excessive cell infusion could lead to immune activation and poor retention. Further research should also focus on optimizing the dose response relationship and route of administration [34]. ACCRUE 2015 meta-analysis by Gyongyosi et al. which includes trials involving first generation stem cells found no significant benefits in improving cardiac function [35]. In contrast, Afzal et al. suggests significant improvements [36]. These conflicting findings emphasize further research to eliminate the factors contributing to discrepancies in outcomes.

The inconsistent clinical evidence regarding first generation stem cell therapies has shifted the focus of research towards second generation therapies that include cardiac derived cells (CDCs), cardiopoietic MSCs (cpMSCs) and induced pluripotent stem cells (iPSCs). Pre-clinical and clinical trials such as CHART-1 trial [37] represent that cpMSCs improve LVEF, but the changes were not statistically significant, however, they improved quality of life in patients treated with stem cell therapy. The C-CURE trial [38] also suggests that cpMSCs not only improve LVEF but also improve quality of life and event free survival. Safety of cpMSCs has been established but there is still room to optimize its therapeutic efficacy. The CADUCEUS trial [39] is the only clinical study to date that has demonstrated significant improvements in LVEF and increased viable myocardium [40]. Meta analysis by Vo et al. 2024 also showed favorable improvements in LVEF and reductions in scar size [41].

While the focus of most clinical trials has been on cardiomyocyte regeneration, it is very important to recognize the role of non-cardiomyocyte factors in cardiac repair [42]. Endothelial cells, macrophages, fibroblasts and other immune cells play a crucial role in extracellular matrix remodeling, resolution of inflammation and angiogenesis. Pre-clinical studies such as Quaife-Ryan et al. and Li et al. suggest that endothelial cells and T regulatory cells can enhance cardiomyocyte regeneration by increasing angiogenesis and facilitating cardiomyocyte proliferation respectively [43, 44].

Stem cell therapies show promising results but the long-term durability and benefits of these therapies along with their safety profile needs further investigation. Furthermore, the heterogeneity necessitates the differences in the trials such as delivery methods, time of administration, dosage and patient characteristics need to be studied to optimize therapy.

Future therapies could benefit from targeting these non-cardiomyocyte factors and optimizing second generation stem cell therapies through gene editing such as CRISPR-Cas9, bioengineering such as hydrogel based delivery systems, genetic modification of stem cells by expressing angiogenic factors such as VEGF and IGF-1 and use of AI driven analytics to monitor or predict response of each patient to stem cell therapy so that tailored treatment could be provided [45, 46]. 

Some studies have reported improved LVEF after cell-based therapies at early follow-up. However, such evidence is of low quality, and the results were not of information size to yield robust conclusions regarding the overall interpretation of these findings [47]. Furthermore, inconsistent outcome measurements make it even more challenging to interpret results from different trials. Considering these challenges, a holistic assessment of the effects of cardiomyocyte regeneration therapies on major parameters, specifically LVEF, scar size, and infarct size, is therefore necessary.

There is a need for detailed knowledge regarding the efficacy and risks associated with cardiomyocyte regeneration therapies to optimize treatment strategies for patients with ischemic heart disease. The results of this study will further guide future research and inform clinical decision making to address the imperative need for therapies that can reverse or mitigate myocardial damage.

## Conclusion

Our meta-analysis demonstrates moderate but statistically significant improvements in LVEF and reduction in scar size with stem cell therapies. However, the heterogeneity among studies calls for the need of standardized protocols in stem cell preparation, delivery, dosage and patient characteristics to ensure consistent outcomes. Future studies should focus on optimizing second generation stem cell therapies which include iPSCs, cpMSCs, CDCs by integrating non cardiomyocyte mechanisms such as immune modulation and angiogenesis to increase therapeutic efficacy. To achieve more robust improvements, genetic modifications using genetic engineering and bioengineering techniques should also be explored.

## Data Availability

Data was extracted from different databases and all the authors made sure that they are compliant to the privacy policy.
